# Granuloma Formation in a *Cyba*-Deficient Model of Chronic Granulomatous Disease Is Associated with Myeloid Hyperplasia and the Exhaustion of B-Cell Lineage

**DOI:** 10.3390/ijms22168701

**Published:** 2021-08-13

**Authors:** Rodrigo Prieto-Bermejo, Marta Romo-González, Alejandro Pérez-Fernández, María Carmen García-Macías, Carmen Sánchez-Bernal, Ignacio García-Tuñón, Jesús Sánchez-Yagüe, Manuel Sánchez-Martín, Ángel Hernández-Hernández

**Affiliations:** 1 Departamento de Bioquímica y Biología Molecular, Universidad de Salamanca, 37007 Salamanca, Spain; rodrix@usal.es (R.P.-B.); martarogo@usal.es (M.R.-G.); alexpf@usal.es (A.P.-F.); csabe@usal.es (C.S.-B.); sanyaj@usal.es (J.S.-Y.); 2 IBSAL (Instituto de Investigación Biomédica de Salamanca), 37007 Salamanca, Spain; adolsan@usal.es; 3 Unidad de Patología Molecular Comparada, Centro de Investigación del Cáncer-IBMCC (USAL-CSIC), 37007 Salamanca, Spain; janagm@usal.es; 4 Unidad de Diagnóstico Molecular y Celular del Cáncer, Centro de Investigación del Cáncer-IBMCC (USAL-CSIC), 37007 Salamanca, Spain; ignacio.tunon@usal.es; 5 Servicio de Transgénesis, Nucleus, Universidad de Salamanca, 37007 Salamanca, Spain; 6 Departamento de Medicina, Universidad de Salamanca, 37007 Salamanca, Spain

**Keywords:** chronic granulomatous disease, inflammation, immune, murine model, myeloproliferative disease, NADPH oxidase

## Abstract

Haematopoiesis is a paradigm of cell differentiation because of the wide variety and overwhelming number of mature blood cells produced daily. Under stress conditions, the organism must adapt to a boosted demand for blood cells. Chronic granulomatous disease (CGD) is a genetic disease caused by inactivating mutations that affect the phagocyte oxidase. Besides a defective innate immune system, CGD patients suffer from recurrent hyper-inflammation episodes, circumstances upon which they must face emergency haematopoiesis. The targeting of *Cybb* and *Ncf1* genes have produced CGD animal models that are a useful surrogate when studying the pathophysiology and treatment of this disease. Here, we show that *Cyba^−/−^* mice spontaneously develop granuloma and, therefore, constitute a CGD animal model to complement the existing *Cybb^−/−^* and *Ncf1^−/−^* models. More importantly, we have analysed haematopoiesis in granuloma-bearing *Cyba^−/−^* mice. These animals showed a significant loss of weight, developed remarkable splenomegaly, bone marrow myeloid hyperplasia, and signs of anaemia. Haematological analyses showed a sharped decrease of B-cells and a striking development of myeloid cells in all compartments. Collectively, our results show that granuloma inflammatory lesions dramatically change haematopoiesis homeostasis. Consequently, we suggest that besides their defective innate immunity, the alteration of haematopoiesis homeostasis upon granuloma may contribute to the dismal outcome of CGD.

## 1. Introduction

Chronic granulomatous disease (CGD) is the most common inherited disorder affecting the innate immune system [1,2]. CGD is caused by defects in the phagocyte oxidase, a membrane-bound complex that produces huge amounts of reactive oxygen species (ROS) during the respiratory burst, which is required for pathogen clearing [3]. Bacterial and fungal infections affecting airways, skin, gastrointestinal tract, lymph nodes, liver, brain, and bones can be life-threatening for CGD patients [2,4]. The severity of the disease varies among patients, and it seems that residual oxidase activity appears to improve patient survival [5]. Most patients develop symptoms early and are diagnosed during the first 5 years of life. Historically CGD was considered “a fatal granulomatous disease of childhood”, with a life expectancy below 10 years [6]. The advances in the management of the disease, including earlier diagnosis, but especially antifungal and antibiotic prophylaxis, have improved the prospects for CGD patients. Nonetheless, the current life expectancy is around 30–40 years, with a quality of life that inversely correlates with age [7,8,9].

Under stress situations, such as infection or inflammation, the organism switches from “steady-state haematopoiesis” to “emergency haematopoiesis” [10], an issue that has become a priority in the field [11]. An intriguing aspect of CGD is its association with hyper-inflammation phenomena, like granuloma, which is present in ≥50% of CGD patients [9] and gives name to this disease [12,13]. These lesions might arise from defective pathogen clearing [14] and spontaneously as well. Studies in CGD animal models challenged with *Aspergillus* suggest that the observed lethal outcome is caused by hyper-inflammation rather than aspergillosis [15], which highlights the relevance of inflammation on CGD pathophysiology [16]. It is thus conceivable that CGD patients must face emergency haematopoiesis frequently. Therefore, better comprehension of how the haematopoietic system of CGD patients responds to this challenge can help address the underlying mechanisms of this regulation.

Around 70% of CGD cases are due to mutations in the NOX2 coding gene, *CYBB*. NOX2 or gp91^phox^ is the catalytic subunit of the complex and the founding member of the NADPH oxidase family, which is comprised of seven isoforms in mammals (NOX1-NOX5, DUOX1 and DUOX2) [3]. CGD can also be triggered by mutations in genes encoding other subunits like *NCF1* (p47^phox^), *CYBA* (p22^phox^) and *NCF2* (p67^phox^), accounting for 20%, 5% and 5% of cases, respectively [2].

CGD animal models, generated by targeting *Cybb* [17] or *Ncf1* [18] genes, have been very useful for the study of the pathophysiology and treatment of this disease [19]. The nmf33 mouse strain displays a missense mutation in the *Cyba* gene, which seems to eliminate p22^phox^ protein expression. In addition, nmf33 mice do not show superoxide production by phagocytes and are susceptible to airway bacterial infections [20]. More recently, it has been reported that the same mouse strain is very prone to severe colitis [21]. Bearing this in mind, it could be surmised that the deletion of the *Cyba* gene would provide us with an alternative CGD animal model, but surprisingly this has not been formally proven. Unlike Nox2 and p47^phox^, which only appear in the Nox2 complex, p22^phox^ is present in four different NADPH oxidases isoforms, and, therefore, removing p22^phox^ may not necessarily have the same effect as removing Nox2 and p47^phox^. In summary, it would be interesting to add a *Cyba*^−/−^ CGD model, complementing the existing *Cybb^−/−^* and *Ncf1^−/−^* models.

We have recently generated a *Cyba*^−/−^ model through CRISPR/Cas9 editing technology [22]. Here, we report that these mice spontaneously developed granuloma and, therefore, constitute a CGD animal model, as expected. More importantly, we have analysed the haematopoiesis in granuloma-bearing mice. Under these circumstances, mice showed splenomegaly, a sharp decrease in B-cell lineage, and a hyper-differentiation of myeloid cells, resembling an MPD-like leukaemia (myeloproliferative-disease-like leukaemia). Our results show that granuloma inflammation lesions dramatically change haematopoiesis homeostasis. Consequently, we suggest that besides their defective innate immunity, the alteration of haematopoiesis homeostasis upon granuloma may contribute to the dismal outcome of this disease.

## 2. Results

### 2.1. Cyba^−/−^ Mice Spontaneously Develop Abscesses

**Figure 1:**
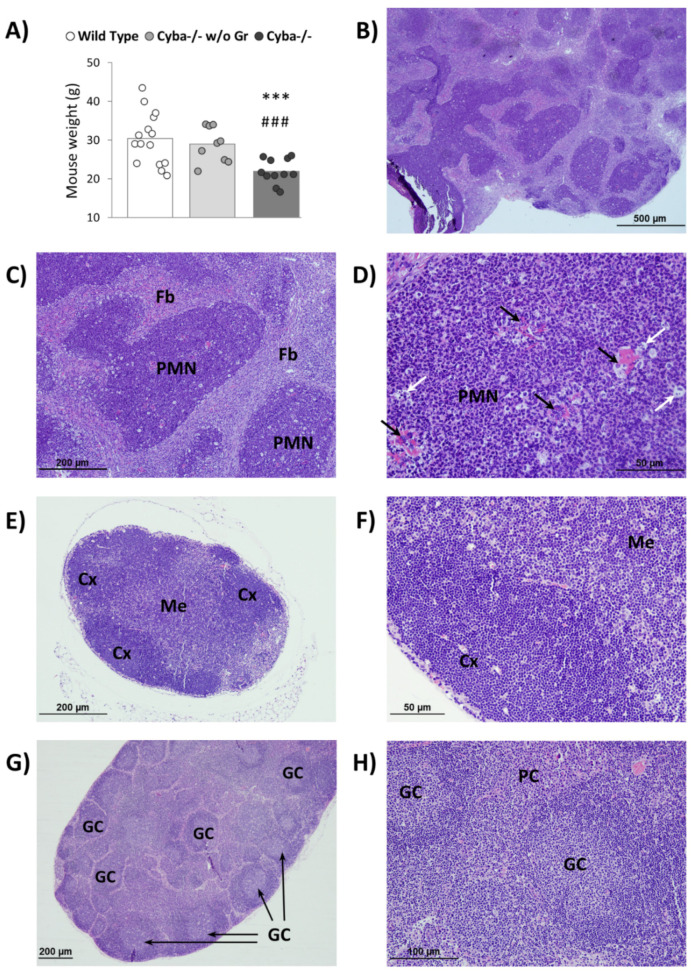
*Cyba^−/−^* mice spontaneously developed abscesses. (**A**) Difference in weight between wild-type (n = 15), *Cyba^−/−^* mice without granuloma (*Cyba^−/−^* w/o Gr; n = 9) and granuloma-bearing *Cyba^−/−^* mice (*Cyba^−/−^*; n = 11). *** *p* < 0.001 vs. control; ^###^ *p* < 0.001 vs. *Cyba^−/−^* mice without granuloma. (**B**–**D**) Haematoxylin–eosin staining of an abscess originated in the muzzle of a *Cyba^−/−^* mouse. PMN: polymorphonuclear leukocytes. Fb: fibroblasts. Black arrows: bacteria. White arrows: macrophages. (n = 8). (**E**–**H**) Haematoxylin–eosin staining of submaxillary lymph nodes in control (**E**,**F**) and granuloma-bearing *Cyba^−/−^* (**G**,**H**) mice. Cx: cortex. Me: medulla. GC: germinal centres. PC: plasma cells. (n = 4). Magnification: 4× (**B**), 10× (**C**), 40× (**D**), 10× (**E**), 32× (**F**), 5× (**G**), 20× (**H**).

We have recently described the generation of a *Cyba*^−/−^ mouse model using CRISPR/Cas9 technology [22]. Similar to what has been reported before for Nox2-deficient mice [23], despite being housed in a specific pathogen-free (SPF) environment, a significant number of *Cyba*^−/−^ mice (around 30% of males and almost 70% of females) spontaneously developed abscesses. These lesions were mostly found in the mouth and gastrointestinal tract (Figure S1A) and were associated with a significant loss of body weight (Figure 1A), suggesting a life-threatening situation. In the absence of granuloma, *Cyba*^−/−^ mice did not show differences in body weight with respect to control animals (Figure 1A).

Histological analyses revealed that these abscesses were composed mainly of polymorphonuclear (PMN) leukocytes encapsulated by fibroblasts (Figure 1B,C and Figure S1B,C). The presence of bacteria colonies (Figure 1D, black arrows) suggested that this could be the trigger of granuloma.

Additionally, granuloma-surrounding lymph nodes were notoriously swollen, with abundant germinal centres (GC), accompanied by plasma cell hyperplasia (Figure 1E–H). Moreover, micro-abscesses of PMN leukocytes surrounded by fibroblasts could also be observed within the lymph nodes (Figure S1D–G).

### 2.2. Cyba^−/−^ Mice Bearing Abscesses Display Splenomegaly and Bone Marrow Myeloid Hyperplasia

**Figure 2:**
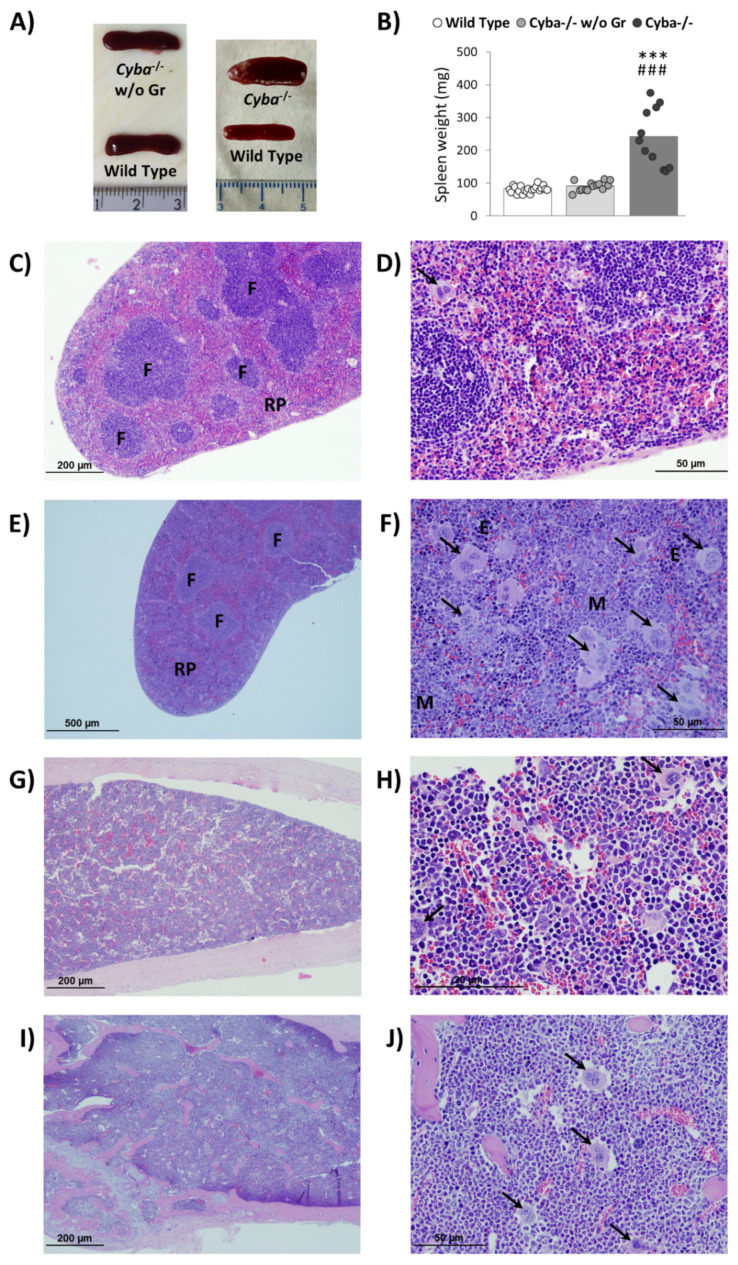
*Cyba^−/−^* mice with abscesses showed splenomegaly and myeloid hyperplasia. (**A**) Spleen of control mice, *Cyba^−/−^* mice without granuloma (*Cyba^−/−^* w/o Gr) and granuloma-bearing *Cyba^−/−^* mice (*Cyba^−/−^*). (**B**) Difference in weight of spleen between *Cyba^−/−^* mice without granuloma (*Cyba^−/−^* w/o Gr; n = 14), granuloma-bearing *Cyba^−/−^* mice (*Cyba^−/−^*; n = 11), and control mice (n = 21). *** *p* < 0.001 vs. control; ^###^ *p* < 0.001 vs. *Cyba^−/−^* mice without granuloma. (**C**–**F**) Haematoxylin–eosin staining of spleens in control (**C**,**D**) and granuloma-bearing *Cyba^−/−^* (**E**,**F**) mice. RP: red pulp. F: white pulp follicles. M: myeloid cells. E: erythroid cells. Black arrows: megakaryocytes. (n = 4). (**G**–**J**) Haematoxylin–eosin staining of femur bone marrow in control (**G**,**H**) and granuloma-bearing *Cyba^−/−^* (**I**,**J**) mice. Black arrows: megakaryocytes. (n = 3). Magnification: 8× (**C**), 40× (**D**), 4× (**E**), 40× (**F**), 8× (**G**), 60× (**H**), 8× (**I**), 40× (**J**).

Abscess-bearing mice showed significant splenomegaly versus control animals (Figure 2A,B), thus suggesting boosted haematopoiesis in this organ. Histological analyses revealed that this alteration was mainly due to red pulp expansion, which was highly disorganised as well. This loss of structure was accompanied by myeloid and erythroid hyperplasia and an unusual increase in megakaryocyte numbers (Figure 2C–F and Figure S2A–C). Interestingly, in the absence of granuloma, *Cyba*^−/−^ mice did not show splenomegaly (Figure 2A,B), and histological analyses showed no alterations (data not shown).

On the other hand, BM myeloid hyperplasia with PMN leukocytes as the predominant cell type, as well as a high number of megakaryocytes and scarce lymphocytes, were observed in CGD mice bearing granuloma versus their healthy counterparts (Figure 2G–J and Figure S2D–F).

In contrast to the alterations found in spleen and BM upon granuloma formation, *Cyba*^−/−^ mice thymus did not display remarkable changes over wild-type mice (Figure S3).

### 2.3. Cyba^−/−^ Mice Bearing Abscesses Undergo Drastic Changes in Haematopoiesis Homeostasis

**Figure 3:**
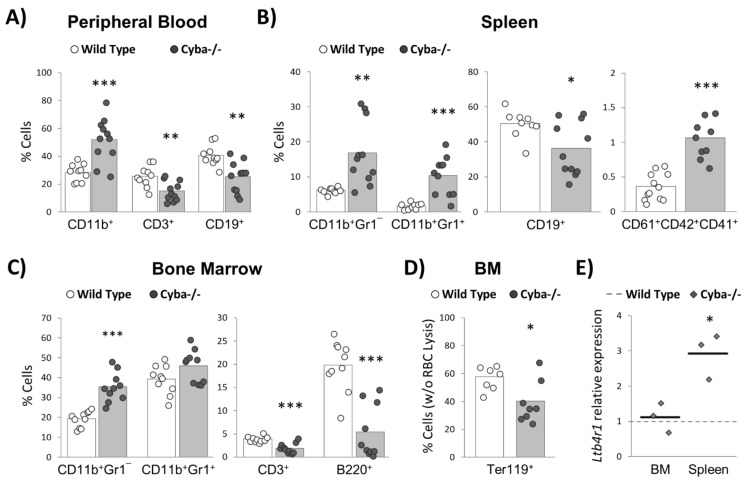
Changes in mature haematopoietic lineages in granuloma-bearing *Cyba^−/−^* mice. Percentage of haematopoietic populations in peripheral blood (PB) (**A**), spleen (**B**) and bone marrow (BM) (**C**,**D**) of *Cyba^−/−^* and control mice. CD11b^+^: myeloid populations; CD11b^+^Gr1^−^: monocytic populations; CD11b^+^Gr1^+^: granulocytic populations; CD3^+^: T-lymphocytes; CD19^+^ and B220^+^: B-lymphocytes; CD61^+^CD42^+^CD41^+^: megakaryocytes; Ter119^+^: erythroid populations. (**E**) Relative expression of the *Ltb4r1* gene in the BM and spleen of *Cyba^−/−^* (diamond dots) mice with respect to control mice (discontinuous line), analysed by qRT-PCR. Black lines represent the group mean. * *p* < 0.05, ** *p* < 0.01, *** *p* < 0.001.

We next analysed haematopoietic lineages in peripheral blood (PB), spleen, and BM. Compared to wild-type animals, *Cyba*^−/−^ mice bearing abscesses showed a significant increase in CD11b^+^ myeloid cells and a decrease in CD3^+^ and CD19^+^ lymphoid cells in PB (Figure 3A). These alterations would be attributable to the inflammatory process associated with CGD, as no differences between wild-type and granuloma-free *Cyba*^−/−^ mice were found in our previous work [22].

Next, spleens of granuloma-bearing *Cyba*^−/−^ mice showed a significant increase of myeloid cells, namely, monocytes (CD11b^+^Gr1¯) and granulocytes (CD11b^+^Gr1^+^), and a concomitant decrease of CD19^+^ B-cells (Figure 3B). In addition, the proportion of megakaryocytes was significantly higher in granuloma-bearing *Cyba*^−/−^ mice (Figure 3B). Overall, these results confirmed the myeloid hyperplasia and the increased megakaryocyte numbers observed in the histological analyses (Figure 2).

Consistently, granuloma-bearing *Cyba*^−/−^ mice displayed an increase in myeloid cells and a decrease in T- and B-lymphocytes in the BM (Figure 3C). Moreover, we observed a significant reduction in erythroid cells (Figure 3D), which, together with the noticeable paler colour shown by the cell pellet collected from the BM (Figure S4) and the reduced presence of mature erythrocytes seen in the histological sections of the BM and spleen (Figure 2), was strong evidence of anaemia.

It has recently been shown that the overproduction of leukotriene B4 (LTB4) by CGD neutrophils contributes to hyper-inflammation and abscess formation [24]. Moreover, LTB4 can induce *in vitro* myeloid differentiation [25]. In this regard, we found a significant upregulation of the gene-encoding LTB4 receptor (*Ltb4r1* or BLT1) in the spleen of granuloma-bearing *Cyba^−/−^* mice (Figure 3E), thus suggesting that LTB4-BLT1 signalling could be contributing to inflammation and myelopoiesis in this organ.

In summary, granuloma-bearing *Cyba^−/−^* mice show a haematopoietic differentiation skewed towards myeloid lineage, detrimental to lymphoid differentiation.

### 2.4. Granuloma Induces a Sharp Decrease in Haematopoietic Progenitor Cells 

**Figure 4:**
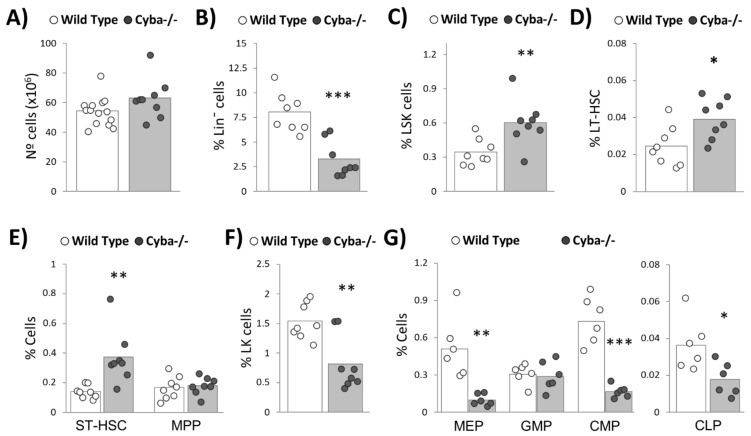
Granuloma-bearing *Cyba^−/−^* mice show a reduction in haematopoietic progenitors. (**A**) Total number of bone marrow (BM) cells in *Cyba^−/−^* and control mice after red blood cell (RBC) lysis. (**B**,**C**) Percentage of Lin^−^ and Lin^−^Sca-1^+^c-Kit^+^ (LSK) cells in the BM of *Cyba^−/−^* and control mice, respectively. (**D**,**E**) Analysis of subpopulations of haematopoietic stem cells (HSCs) in the BM of *Cyba^−/−^* and control mice. LT-HSC: long-term HSC (LSK CD34^−^Flt3^−^). ST-HSC: short-term HSC (LSK CD34^+^Flt3^−^). MPP: multipotent progenitors (LSK CD34^+^Flt3^+^). (**F**) Percentage of Lin^−^Sca-1^−^c-Kit^+^ (LK) cells in the BM of *Cyba^−/−^* and control mice. (**G**) Analysis of subpopulations of haematopoietic progenitor cells (HPCs) in the BM of *Cyba^−/−^* and control mice. MEP: megakaryocyte-erythrocyte progenitors (LK CD34^−^CD16/32^−^). GMP: granulocyte–monocyte progenitors (LK CD34^+^CD16/32^+^). CMP: common myeloid progenitors (LK CD34^+^CD16/32^−^). CLP: common lymphoid progenitors (Lin^−^Sca-1^low^c-Kit^low^IL-7Rα^+^). * *p* < 0.05, ** *p* < 0.01, *** *p* < 0.001.

*Cyba^−/−^* mice developing granuloma displayed no significant alteration in total BM cell numbers versus their wild-type counterparts (Figure 4A). Conversely, they showed a sharp decrease in the percentage of Lin¯ cells (Figure 4B), thus suggesting an alteration of the haematopoietic stem and progenitor cell (HSPC) compartment. Strikingly, Lin¯Sca-1^+^c-Kit^+^ (LSK), long-term (LT-HSC) and short-term haematopoietic stem cell (ST-HSC) populations were significantly overrepresented in *Cyba^−/−^* mice (Figure 4C–E). It is worth noting that this increase cannot be attributed to granulomatous lesions, as this has been previously observed in the absence of inflammatory alterations [22]. Of interest, a significant decrease in Lin¯Sca-1¯c-Kit^+^ (LK) (Figure 4F), megakaryocyte-erythrocyte progenitors (MEP), common myeloid progenitors (CMP) and common lymphoid progenitors (CLP) (Figure 4G) was observed in granuloma-bearing *Cyba*^−/−^ mice. This is in contrast with our previous results in granuloma-free *Cyba*^−/−^ mice, which did not show this difference versus wild-type animals [22]. In summary, CGD-associated inflammation induced a sharp decrease in Lin¯ cells, which could be explained by the exhaustion of committed progenitor populations.

### 2.5. Granuloma Alters B-Cell Differentiation 

**Figure 5:**
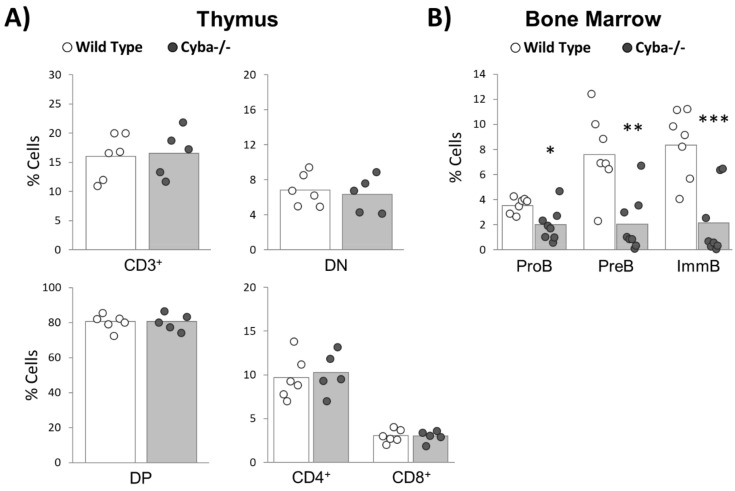
Granuloma-bearing *Cyba^−/−^* mice show an alteration in the differentiation of B-cells. (**A**) Analysis of T-lymphocyte differentiation stages in the thymus of *Cyba^−/−^* and control mice. DN: double-negative cells (CD4¯CD8¯). DP: double-positive cells (CD4^+^CD8^+^). (**B**) Analysis of B-lymphocyte differentiation stages in the bone marrow (BM) of *Cyba^−/−^* and control mice. ProB: B220^+^CD43^+^IgM^−^. PreB: B220^+^CD43^−^IgM^−^. ImmB: (immature B-cell) B220^+^CD43^−^IgM^+^. * *p* < 0.05, ** *p* < 0.01, *** *p* < 0.001.

Whereas a slight decrease in CD3^+^ T-lymphocytes in SP and BM had been observed in *Cyba^−/−^* mice developing granuloma (Figure 3A,C), no significant differences in total CD3^+^, early differentiation compartments (DN: double-negative cells (CD4¯CD8¯). DP: double-positive cells (CD4^+^CD8^+^)) or CD4^+^ and CD8^+^ cells were found in the thymus (Figure 5A). Consistently, morphological alterations were also absent at the histologic level in this organ (Figure S3). In summary, T-cell differentiation was not impaired by granuloma development in *Cyba*-deficient mice.

According to histological (Figure 2G–J) and flow cytometry analyses (Figure 3A–C), granuloma-bearing mice showed a significant decrease in B-cells. In agreement with that, these mice also showed a strong decrease in different stages throughout B-cell maturation (proB, preB and immature B-cells) (Figure 5B).

## 3. Discussion

The maintenance of steady-state haematopoiesis requires the production of an overwhelming amount of mature blood cells on a daily basis. Moreover, under stress conditions, such as infections, bleeding or inflammation, the haematopoietic system must fulfil an enhanced demand of mature blood cells [10]. Stress-induced haematopoiesis must be tightly controlled to avoid an aberrant activation, which could lead to chronic diseases or cancer. Therefore, unravelling the regulatory mechanisms of emergency haematopoiesis has become a priority for haematologists [11].

Despite the improvements in prophylaxis and the access to antibiotics, life expectancy and quality of life for CGD patients remains poor [7,8,9], in part due to their inability to resolve the associated hyper-inflammation [9,15]. Therefore, a reasonable assumption is that CGD patients must regularly face emergency haematopoiesis. A deeper comprehension of how this happens and the consequences for CGD patients is, therefore, paramount to further decipher the pathophysiology of this disease.

CGD in humans is triggered by mutations in *CYBB*, *NCF1*, *NCF2* and *CYBA* [26]. The first three genes encode proteins exclusively belonging to the NOX2 complex. In contrast, p22^phox^, the *CYBA* gene product, is essential for four different NADPH oxidases [3]. The targeting of *Cybb* [17] or *Ncf1* [18] has provided mouse models that represent a good surrogate for studying CGD pathophysiology. Although it could have been expected that the deletion of the *Cyba* gene would produce an alternative CGD model, this possibility has not been formally tested. According to our previous data [22], haematopoietic progenitor cells express three p22^phox^-dependent NADPH oxidases (Nox1, Nox2 and Nox4). Therefore, p22^phox^ mutations would affect the activity of all of them. Moreover, p22^phox^ mutations might affect other biological phenomena relaying on Nox3, such as balance control [20]. Bearing all this in mind, a *Cyba*-deficient model would faithfully mimic the pathophysiology of human patients showing *CYBA* mutations.

Here, we show that *Cyba^−/−^* mice spontaneously develop granuloma and, consequently, represent an alternative CGD animal model to the ones previously reported [17,18]. The proportion of *Cyba^−/−^* mice developing granulomas, around 50%, is similar to that described in human patients [9]. However, a striking feature was that *Cyba^−/−^* female mice were more susceptible to developing granuloma than males. It has been reported before that the CGD inflammatory manifestation can also be observed in X-linked female CGD carriers [26]. It might be possible that CGD female patients are more prone to developing inflammatory responses, which seems an interesting notion to be investigated in the future. We speculate that the action of oestrogens may be an underlying mechanism of this higher susceptibility [27].

We have previously shown that *Cyba*^−/−^ mice present the enrichment of haematopoietic stem cell populations due to enhanced cell proliferation [22]. Similar results have been reported by other authors upon *Cybb* deletion [28]. These results support the importance of NADPH oxidase activity for in vivo haematopoiesis. However, to comprehend the implication of p22^phox^ in haematopoiesis, it would be necessary to consider the activity of Nox1 and Nox4 in this context [22].

Here, we have analysed whether granuloma inflammatory lesions alter haematopoiesis homeostasis in our *Cyba*-based CGD model. As CGD patients often present sterile inflammatory processes [29,30,31], we used *Cyba^−/−^* mice that spontaneously developed granuloma inflammatory lesions as a model instead of animal challenging with pathogens. 

In our previous report, *Cyba*^−/−^ mice showing hyper-inflammation symptoms were not included [22]. This provided us with an opportunity to dissect the effect of inflammation on CGD haematopoiesis. Our analyses support that inflammation will not alter the HSC compartment in our CGD mouse model. However, there is a pronounced decrease of Lin¯ progenitor cells, which suggests that emergency haematopoiesis is sustained by an increase in the activity of the HSPC compartment. Granuloma formation triggered pronounced monocytosis and neutrophilia, which could be explained by the enhanced activity of CMP.

In contrast, the reduction of CLP was not accompanied by an increase in lymphoid lineages, rather the opposite, a significant reduction of B-cells. It has been proposed that under stress haematopoiesis, there is strong competition between myelopoiesis and lymphopoiesis for access to the growth factors present in the BM [10,32]. This would lead to the mobilisation of lymphoid cells to peripheral tissues such as the spleen [33]. Upon inflammation, *Cyba*^−/−^ mice displayed splenomegaly, together with enhanced haematopoietic activity in the spleen, but a significant reduction in the proportion of B-cells. Therefore, competition between lymphopoiesis and myelopoiesis would be skewed towards the latter in this organ, as is the case of the BM. In summary, the spleen does not compensate for the lack of B-cell development observed at the BM. B-cell progenitors are very sensitive to apoptosis, and pro-inflammatory cytokines can induce B-cell progenitor cell death [34], which could explain the reduction of these cells observed in CGD mice upon granuloma appearance. Moreover, inflammatory cytokines induce the differentiation of B-cells into plasma cells [35]. Lymph nodes of granuloma-bearing *Cyba*^−/−^ mice presented an unusually high number of germinal centres and plasma cell hyperplasia. B-cells migration to lymph nodes and differentiation into plasma cells could explain, at least in part, the depletion of the B-cell lineage.

Depletion of CLP could affect both B- and T-lineages. However, although a slight decrease in T-cells was observed in the PB and BM, no significant differences in this lineage were observed in the thymus between granuloma-bearing *Cyba*^−/−^ mice and control mice. This suggests that inflammation in this scenario especially alters B-cell lineage. The continuous episodes of inflammation may lead to the exhaustion of B-cell lineage in CGD patients, and we suggest that this factor might contribute to the unfavourable outcome of the disease.

Megakaryocyte differentiation can also be influenced by inflammation [36]. A sub-population of HSC expresses the von Willebrand factor (vWF) and is primed for the production of megakaryocytes and platelets [37]. Haas et al. have recently demonstrated that inflammation can activate this subset of HSC to boost megakaryocyte differentiation [38]. Consistently, we detected an increase in megakaryocytes in our CGD mouse model upon inflammation. The reasons for the enhancement of megakaryopoiesis upon inflammation remain elusive. It has been proposed that platelets could function as antigen-presenting cells. However, the ability of platelets to endocite pathogens remains highly controversial. Another hypothesis is that platelets could enhance the activity of phagocytes, contributing in this way to innate immunity [36]. On the other hand, megakaryocytes not only generate platelets but are also important niche cells for HSCs [39], and, therefore, they could contribute to HSC recovery [40].

Compelling evidence sustains that the chronic activation of HSC is a risk factor for the development of haematological malignancies [10]. The haematological features observed in our CGD mice agree well with MPD-like leukaemia, according to the guidelines provided by Kogan et al. [41]. Moreover, we also observed a reduction of red cells at the BM that could represent the anaemia commonly associated with myeloid neoplasms [42]. While the alterations described in this work for *Cyba^−/−^* mice are a reaction to hyper-inflammation, the continuous challenging of CGD patients’ haematopoietic systems is a dangerous scenario that could lead to leukaemic transformation. In agreement with this hypothesis, epidemiologic studies have revealed a significant association between chronic infections and inflammation and the appearance of myeloid malignancies [43].

In summary, in this work, we report that *Cyba^−/−^* mice develop CGD symptoms and, therefore, represent a novel CGD animal model. Moreover, our results show that granuloma formation is accompanied by the alteration of haematopoiesis homeostasis, leading to splenomegaly, anaemia, myeloid hyperproliferation, the reduction of haematopoietic progenitors and the exhaustion of B-cell lineage. These haematopoietic alterations, besides the defective innate immunity, are also important factors that may contribute to the dismal outcome of untreated CGD.

## 4. Materials and Methods

### 4.1. Reagents

TRI reagent was from Sigma-Aldrich (Madrid, Spain). Antibodies for the detection of murine CD135 (Flt3), CD34, CD16/32, and CD127 (IL-7Rα) were from eBioscience (Barcelona, Spain). The Lineage Cell Depletion Kit for mice, FcR Blocking reagent and murine flow cytometry antibodies (CD3e, CD4, CD8a, CD11b, CD19, CD43, CD45R (B220), CD117 (c-Kit), anti-Gr1, anti-Sca-1, anti-Ter-119, anti-IgM) were from Miltenyi Biotec (Madrid, Spain). Super Script II reverse transcriptase and RNase OUT ribonuclease inhibitor were from Invitrogen and Thermo Fisher Scientific (Madrid, Spain). GoTaq^R^ qPCR master mix was from Promega (Madrid, Spain).

### 4.2. Animals

C57BL/6J mice were from the University of Salamanca Animal Facility Unit (Salamanca, Spain). *Cyba*-deficient mice (*Cyba*^−/−^), previously generated in our laboratory [22], were used as a CGD model. Mice were kept in pathogen-free animal facilities at the University of Salamanca. Only *Cyba*^−/−^ mice showing abscesses (Figure S1A) and a significant loss of weight (Figure 1A) were included in this study. C57BL/6J wild-type mice of the same gender and age were used as controls.

### 4.3. Histological Analysis

Tissues were fixed with 10% formaldehyde and embedded in paraffin. Then, 2 µm tissue sections were stained with haematoxylin and eosin. An Olympus BX51 microscope connected to an Olympus DP70 colour digital camera (Olympus, Hicksville, NY, USA) was used for taking pictures.

### 4.4. Haematopoietic Lineages Analysis

Peripheral blood (PB), bone marrow (BM) and spleen samples were obtained immediately after sacrificing the mice. Except in the specified experiments, red blood cells (RBCs) were lysed with 155 mM ammonium chloride, 10 mM KHCO_3_, and 0.13 mM EDTA, before antibody staining. The lineage-specific markers used were as follows: anti-Ter119 for erythrocytes; anti-Gr1 and CD11b for granulocytes and monocyte/macrophages; CD19 and B220 for B-cells, and CD3 for T-cells. B-cell maturation was analysed in the BM: ProB (B220^+^CD43^+^IgM¯), PreB (B220^+^CD43¯IgM¯) and immature B-cells (B220^+^CD43¯IgM^+^). Stages of T-cell maturation were analysed in the thymus based on markers CD4 and CD8 (DN: CD4¯CD8¯; DP: CD4^+^CD8^+^). BM Lin¯ cell subpopulations were identified as follows: LSK (Lin¯Sca-1^+^c-Kit^+^), LT-HSC (Lin¯Sca-1^+^c-Kit^+^CD34¯Flt3¯), ST-HSC (Lin¯Sca-1^+^c-Kit^+^CD34^+^Flt3¯), MPP (Lin¯Sca-1^+^c-Kit^+^CD34^+^Flt3^+^), LK (Lin¯Sca-1¯c-Kit^+^), MEP (Lin¯Sca-1¯c-Kit^+^CD34¯CD16/32¯), CMP (Lin¯Sca-1¯c-Kit^+^CD34^+^CD16/32¯), GMP (Lin¯Sca-1¯c-Kit^+^CD34^+^CD16/32^+^), and CLP (Lin¯Sca-1^low^c-Kit^low^IL-7Rα^+^). Representative flow cytometry plots are shown in Figure S5.

### 4.5. Quantitative RT-PCR

RNA was extracted with TRI reagent, and cDNA was generated with SuperScript II Reverse Transcriptase. qPCRs were carried out using GoTaq^R^ qPCR Master Mix in a StepOne RealTime PCR system (Applied Biosystems, Madrid, Spain). Analysis of data was performed by the comparative Ct method (ΔΔCt), using *Actb* as the endogenous control [22]. qPCR oligonucleotides for *Ltb4r1* were as follows: sense 5′ GACCCTGGCACTAAGACAGA 3′; antisense 5′ AGAACAATGGGCAACAGAGA 3′.

### 4.6. Statistical Analyses and Data Report

Data are expressed as combined dot-bar graphs; each dot represents an individual, and bars denote the mean value of its group. Data were analysed with SPSS 23 software. Two-tailed unpaired Student’s *t*-tests were used, and differences were considered statistically significant when *p* < 0.05 (*), *p* < 0.01 (**) or *p* < 0.001 (*** or ^###^).

## Data Availability

Data is contained within the article or Supplementary Material.

## Supplementary Materials

The following are available online at https://www.mdpi.com/article/10.3390/ijms22168701/s1.
