# Supplementary material for: Granuloma Formation in a Cyba-Deficient Model of Chronic Granulomatous Disease Is Associated with Myeloid Hyperplasia and the Exhaustion of B-Cell Lineage
Source: Int J Mol Sci. 2021 Aug 13;22(16):8701. doi: 10.3390/ijms22168701 (PMC8395996; doi:10.3390/ijms22168701)
Supplement: Supplementary file 1 [file ijms-22-08701-s001.zip › ijms-1272411-supplementary.pdf]

Figure S1

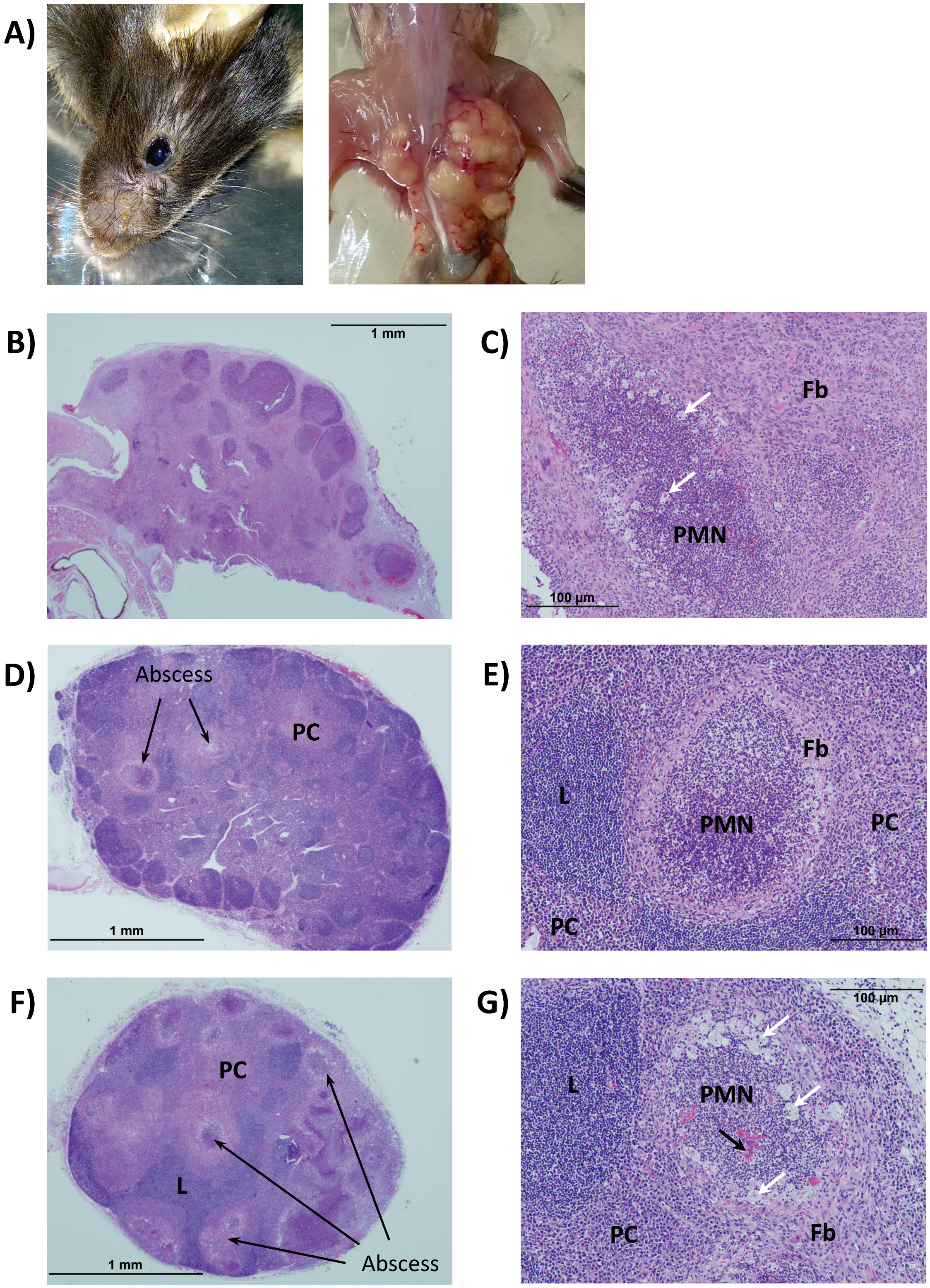

Figure S1. Abscesses developed by *Cyba*$^{-/-}$ mice. A) Images of abscesses appearing in muzzle (left) or in the gastrointestinal tract (right) of *Cyba*$^{-/-}$ mice. **B, C)** Haematoxylin-Eosin staining of an abscess originated in the muzzle of a *Cyba*$^{-/-}$ mice. PMN: Polymorphonuclear leukocytes. Fb: Fibroblasts. White arrows: Macrophages. (n = 8). **D-G)** Haematoxylin-Eosin staining of submaxillary lymph nodes in *Cyba*$^{-/-}$ mice. PC: Plasma cells. L: Lymphoid cells. Black arrow: Bacterial colony. (n = 4). Magnification: 2.5x (B), 20x (C), 3.4x (D, F), 20x (E, G).

Figure S2

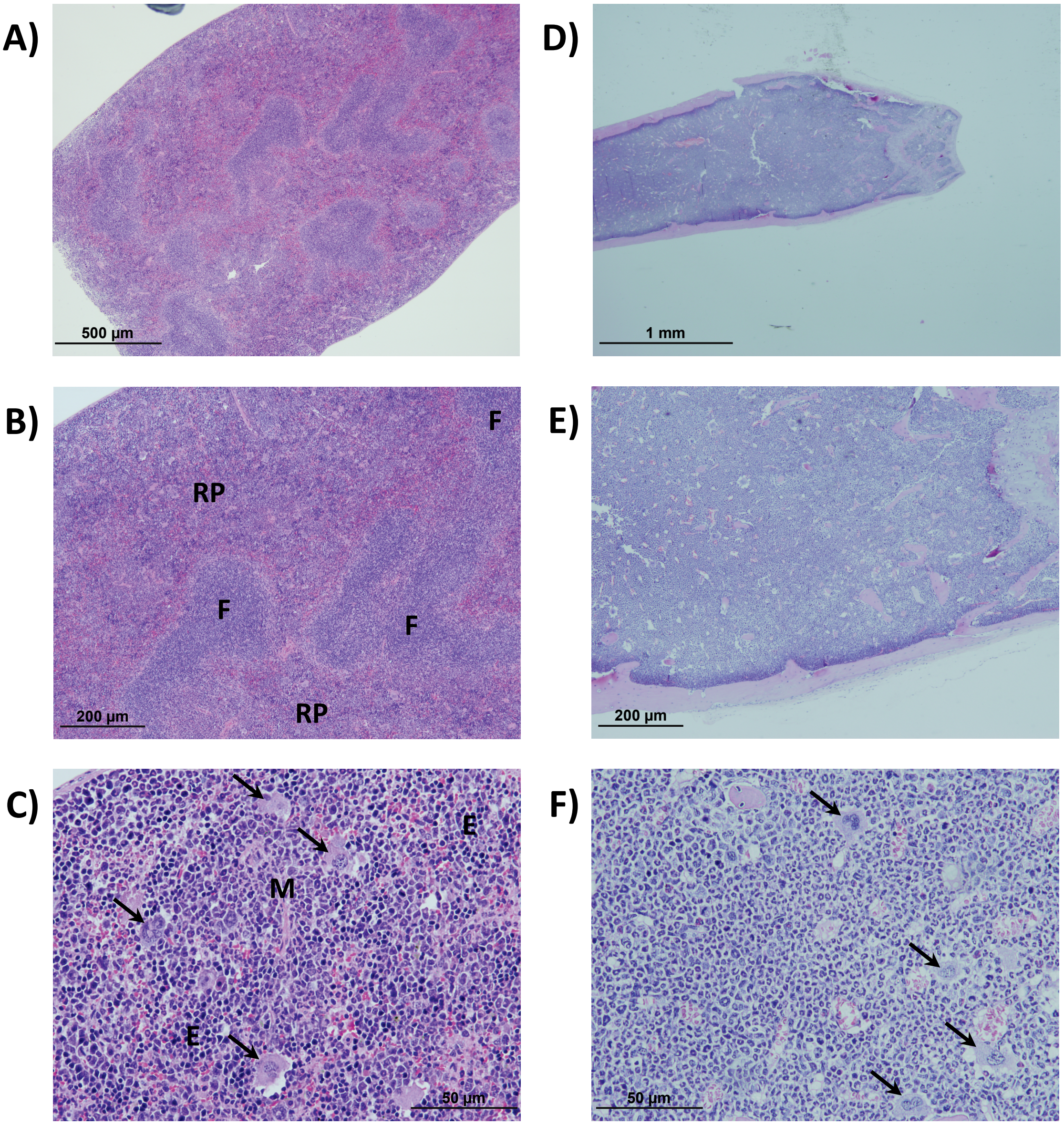

Figure S2. Spleen and bone marrow histological analysis in *Cyba*$^{-/-}$ mice displaying granuloma. The Haematoxylin-Eosin staining of the following organs is shown: **A-C)** Spleen of *Cyba*$^{-/-}$ mice. RP: Red pulp. F: White pulp follicles. M: Myeloid cells. E: Erythroid cells. Black arrows: Megakaryocytes. (n = 4). **D-F)** Femur bone marrow in *Cyba*$^{-/-}$ mice. (n = 3). Magnification: 4x (A), 8x (B), 40x (C), 2.5x (D), 8x (E), 40x (F).

Figure S3

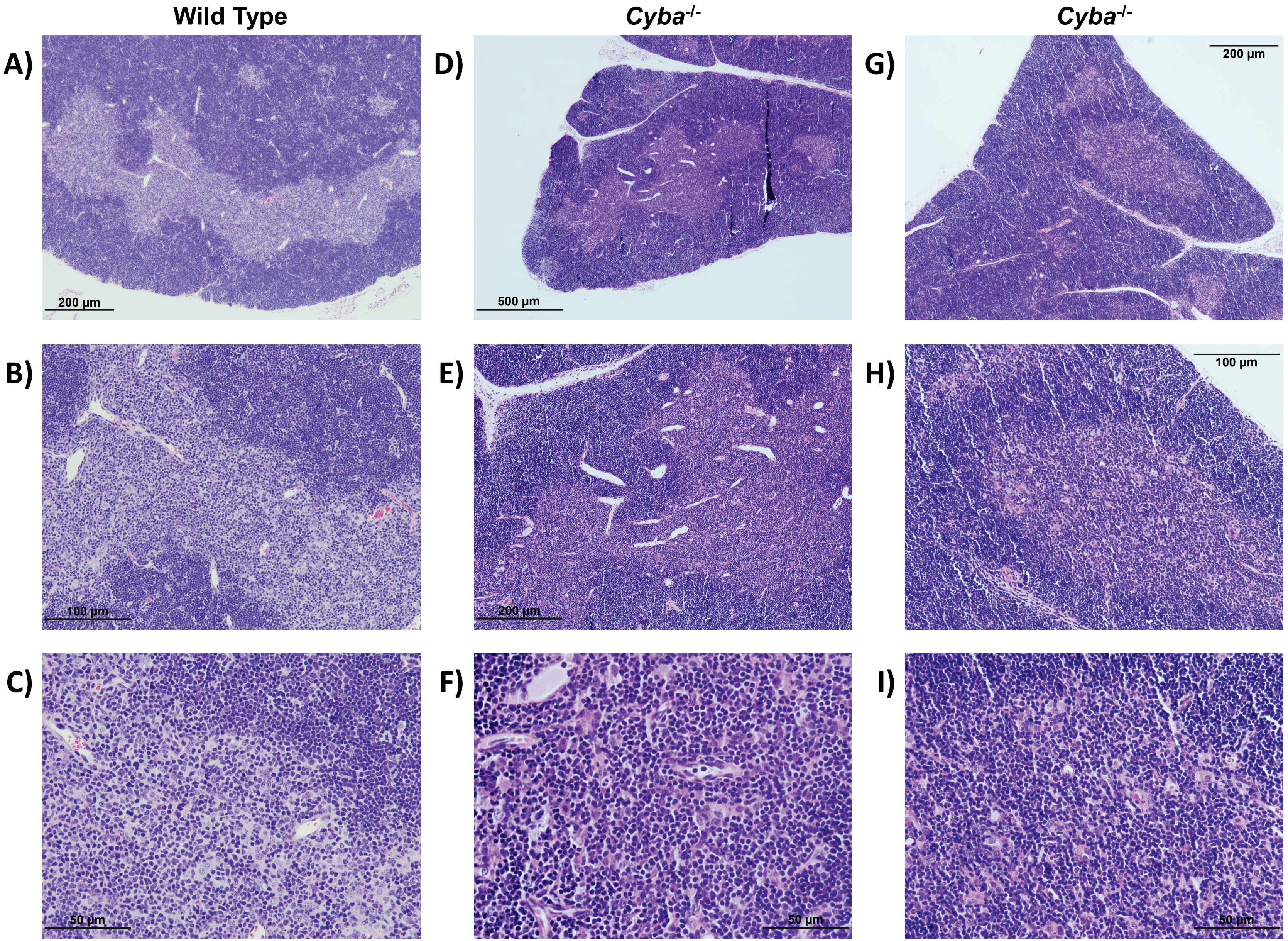

Figure S3. Histological analysis of thymus. The Haematoxylin-Eosin staining of thymus in control **(A, B, C)** and two *Cyba*$^{-/-}$ **(D-F; G-I)** mice is shown. (n = 3). Magnification: 8x (A), 20x (B), 40x (C), 4x (D), 10x (E), 40x (F), 8x (G), 20x (H), 40x (I).

Figure S4

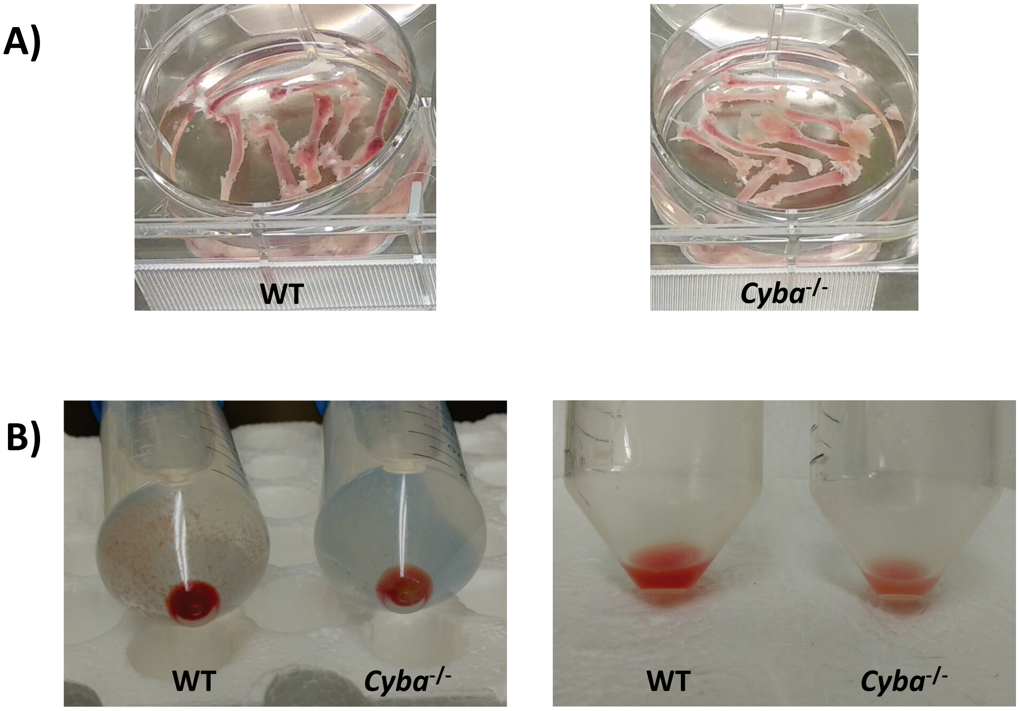

Figure S4. Granuloma-bearing *Cyba*$^{-/-}$ mice bones show a paler colour than control mice. Bones and isolated bone marrow from *Cyba*$^{-/-}$ mice show a paler colour than those of wild type mice. **A)** Bones freshly isolated from wild type and *Cyba*$^{-/-}$ mice. **B)** Bone marrow cells obtained from wild type and *Cyba*$^{-/-}$ mice.

Figure S5

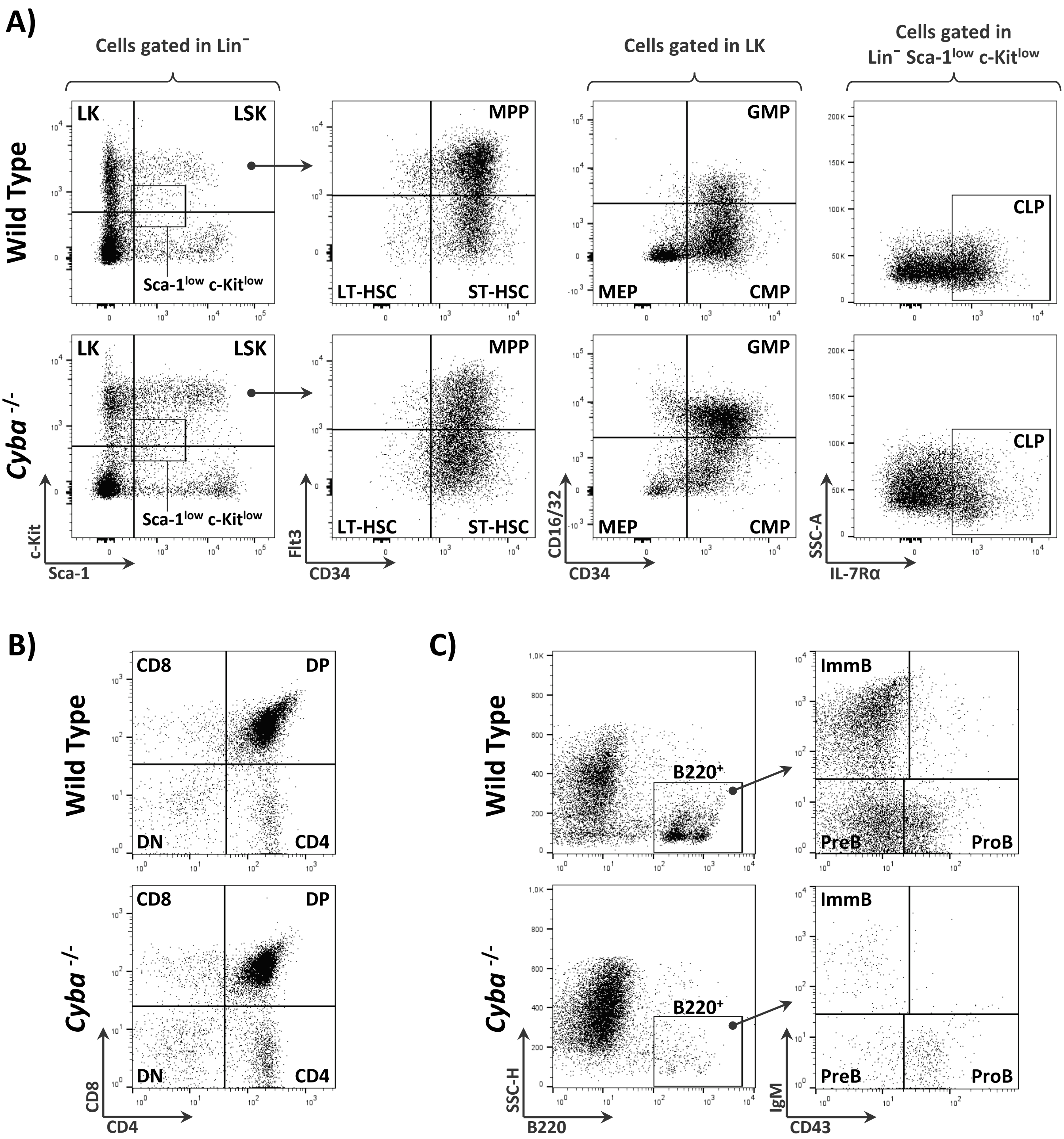

Figure S5. Representative examples of flow cytometry gating for multi-labelled populations. **A)** Haematopoietic stem cell populations in bone marrow from wild type and granuloma-bearing *Cyba*$^{-/-}$ mice. **B)** T lymphocyte populations in thymus, and **C)** pre-B lymphocyte populations in bone marrow from wild type and *Cyba*$^{-/-}$ mice bearing abscesses.
